# Supplementary material for: Identification and functional analysis of non-coding regulatory small RNA FenSr3 in Bacillus amyloliquefaciens LPB-18
Source: PeerJ. 2023 May 15;11:e15236. doi: 10.7717/peerj.15236 (PMC10194069; doi:10.7717/peerj.15236)
Supplement: Supplemental Information 4 [file peerj-11-15236-s004.zip › KO/CK-vs-T1_map/map05165.html]

KEGG PATHWAY: Human papillomavirus infection - Reference pathway


|  |  |
| --- | --- |
| **Human papillomavirus infection - Reference pathway** |  |

[
Pathway menu
| Organism menu
| Pathway entry
| Show description
| User data mapping
]

Human papillomavirus (HPV) is a non-enveloped, double-stranded DNA virus. HPV infect mucoal and cutaneous epithelium resulting in several types of pathologies, most notably, cervical cancer. All types of HPV share a common genomic structure and encode eight proteins: E1, E2, E4, E5, E6, and E7 (early) and L1 and L2 (late). It has been demonstrated that E1 and E2 are involved in viral transcription and replication. The functions of the E4 protein is not yet fully understood. E5, E6, and E7 act as oncoproteins. E5 inhibits the V-ATPase, prolonging EGFR signaling and thereby promoting cell proliferation. The expression of E6 and E7 not only inhibits the tumor suppressors p53 and Rb, but also alters additional signalling pathways. Among these pathways, PI3K/Akt signalling cascade plays a very important role in HPV-induced carcinogenesis. The L1 and L2 proteins form icosahedral capsids for progeny virion generation.

|  |  |  |
| --- | --- | --- |
| Reference pathway | 184% 150% 122% 100% 82% 67% 55% | 图片下载 |
